# Editorial: Case reports in cardiovascular imaging 2023

**DOI:** 10.3389/fcvm.2024.1424893

**Published:** 2024-05-29

**Authors:** Riccardo Liga, Grigorios Korosoglou

**Affiliations:** ^1^ Department of Surgical Pathology, University of Pisa, Pisa, Italy; ^2^ Cardiothoracic and Vascular Department, University Hospital of Pisa, Pisa, Italy; ^3^ Department of Cardiology, Vascular Medicine and Pneumology, GRN Hospital Weinheim, Weinheim, Germany; ^4^ Cardiac Imaging Center Weinheim, Hector Foundation, Weinheim, Germany

**Keywords:** echocardiography, cardiac magnetic resonance (CMR), cardiac computed tomography (CCT), cardiac imaging, interventional cardiology

**Editorial on the Research Topic** Case reports in cardiovascular imaging: 2023

Echocardiography is considered as the “working horse” imaging technique and is used for the assessment of left-ventricular (LV), right-ventricular (RV), and valvular function as well as myocardial strain if required. While echocardiography is a bedside imaging technique, which can be performed without contrast or radiation exposure for the patients, it is dependent on the experience of the operators and on the acoustic windows. CMR, on the other hand, can provide tomographic information of myocardial function, perfusion, viability, and, if required, strain, metabolism, and valvular function, without being limited by the acoustic windows of the patients. CMR is considered the gold-standard for the assessment of chamber dimensions and function as well as the characterization and risk stratification of patients with ischemic heart disease, myocarditis, heart failure, and cardiomyopathy (1–4). Cardiac computed tomography angiography (CCTA) provides isotropic 3D visualization of cardiac structures, including moving coronary arteries with high spatial and temporal resolution and excellent image quality. Additionally, for the visualization of the coronary artery lumen, as provided by invasive coronary angiography, CCTA enables the assessment of the coronary vessel wall, including quantification of plaque volume and composition and assessment of the presence of high-risk coronary plaque features (5, 6), which have been described as pre-cursors of plaque rupture, causing acute coronary syndromes (ACS) (7, 8). Finally, nuclear cardiac imaging techniques allow both the assessment of regional myocardial perfusion (9, 10) and the unique opportunity to perform molecular cardiac imaging with different radiotracers directed towards different molecular targets (11, 12).

Herein, we present and discuss some high-quality case reports from the area of cardiovascular imaging, aiming to enhance its role in clinical practice and contributing to improved diagnostic work-up, patient management, and, potentially, outcomes.

The role of CCTA for guiding PCI is nicely presented in the article by Su et al. on an 83-year-old man with diabetes mellitus, presenting with non-ST-elevation myocardial infarction. CCTA revealed the presence of a single coronary ostium with origin of the left and right coronary artery from the right sinus of Valsalva. The patient was successfully treated with PCI and implantation of several drug-eluting-coronary stents with a good angiographic result.

Coronary artery disease and coronary anomalies are largely represented in our case collection. Thus, the role of CCTA in the diagnosis of coronary artery disease is highlighted in the article by Korosoglou et al., where CCTA detected significant lumen narrowing in a patient with symptomatic coronary artery disease, triggering coronary revascularization by PCI. In addition, CCTA demonstrated attenuated progression of non-calcified atherosclerotic plaque during lipid-lowering treatment of the patient with bempedoic acid, which was attributed to substantial low-density-cholesterol reduction.

Although rare, coronary artery anomalies may have a significant functional and prognostic impact. In this regard, McAlpin et al. reported the case of a 14-year-old boy with a single coronary artery originating from the right coronary sinus. While the suspicion of a coronary anomaly came by transthoracic echocardiography, the final diagnosis required CCTA that also identified the inter-arterial and intramural course of the left coronary artery.

In the article by Oh et al., myocardial perfusion SPECT imaging provided functional information in a 52-year-old woman who had suffered a myocardial infarction with non-obstructive coronary arteries (MINOCA) due to coronary vasospasm at the level of a myocardial bridge, as demonstrated by intravascular ultrasound and CCTA.

Cardiac masses and tumors constitute another important field, where multi-modality cardiac imaging is often required to establish the correct diagnosis (13). The importance of multi-modal imaging in this regard was nicely demonstrated by Li et al. in a very young 23-year-old patient with a primary pericardial sarcoma. Information from CCTA, PET-CT, and CMR, the latter proving detailed visualization of cardiac and extracardiac structures, contributed to timely diagnosis, enabling optimal treatment planning.

Not only is the diagnosis of a cardiac tumor an important domain of cardiac imaging but so too is the differential diagnosis between cardiac tumors and normal anatomical structures of the heart. Thus, in the article by Akiki et al., the authors nicely demonstrated the potential of multi-modality cardiac imaging to establish the diagnosis of left atrial myxoma in a 73-year-old woman presenting with syncope. Echocardiography, CCTA, and CMR were used for pre-operative planning, and 3D printing of the tumor facilitated the use of a robotic approach for surgical removal of the tumor.

When cardiac masses are concerned, multimodality imaging may be particularly helpful for disease characterization and differential diagnosis. In this regard, the article by Kong et al. reported the case of a 69-year-old man with primary invasive cardiac angiosarcoma causing cardiac tamponade, whereby trans-thoracic echocardiography, chest CT, and magnetic resonance imaging were instrumental for tumor staging and for guiding patient management.

The role of multimodality imaging for the diagnosis of cardiac masses is also discussed by Yu et al., reporting on the application of advanced echocardiographic techniques and cardiac CT for the precise characterization of suspected lipomatous atrial septal hypertrophy coupled with atrial septal defect (ASD) in a 68-year-old woman.

In their article, Zhang et al. discussed the case of a 57-year-old woman with obstructive hypertrophic cardiomyopathy treated with “Echocardiography-guided Percutaneous IntraMyocardial Septal Radiofrequency Ablation” (PIMSRA, Liwen procedure). In this case, speckle tracking echocardiography was also able to detect the early improvement of regional cardiac function and the recovery of LV contractile synchronicity following treatment.

The use of nuclear cardiac imaging techniques for disease characterization is reported in two case reports. In the article by Jiang et al., molecular imaging with PET/CT using a ^68^Ga-radiollabeled fibroblast activation protein inhibitor (FAPI) was shown to be able to detect the presence of early activated fibroblasts in a patient with unstable angina and monitor disease activity after coronary revascularization.

The clinical impact of experienced vs. non-experienced operators with echocardiography is nicely demonstrated by Caiati et al. in an 84-year-old woman who was referred to the echocardiographic laboratory for routine examination. The patient had chronic atrial fibrillation and history of heart failure and had been diagnosed with hypertensive heart disease as there was myocardial hypertrophy on the echocardiogram. The experienced view of a senior cardiologist and ultrasound contrast agent administration, however, helped to “unmask” the presence of severe apical hypertrophy of the left ventricle, establishing the correct diagnosis of apical hypertrophic cardiomyopathy.

Finally, the role of multi-modality cardiac imaging using invasive angiography, CCTA, and perfusion mapping of the lungs is nicely demonstrated by Goyal et al. in a 65-year-old man with dyspnea and history of pulmonary embolism 4 years prior to his index clinical presentation. Echocardiography showed RV-dilation with reduced function and CT demonstrated complete occlusion of the right pulmonary artery. Lung perfusion imaging demonstrated some areas of compensated perfusion in the right lung, which originated from coronary collaterals, as demonstrated by invasive coronary angiography. The patient underwent successful pulmonary thromboendarterectomy, which resulted in restoration of lung perfusion and right-ventricular function.

In conclusion, recent advances with non-invasive cardiac imaging are clinically relevant for the diagnostic-work up, timely diagnosis, and subsequent therapeutic management of patients with a multitude of cardiac disorders. All cases reported in our collection represent nice examples that demonstrate how cardiac and, if required, multimodality imaging can be incorporated into daily clinical practice to improve patient care and clinical outcomes.
